# Supplementary figures and images for: Influence of the Chemical Properties of Cereal Grains on the Structure and Metabolism of the Bacteriome of Rhyzopertha dominica (F.) and Its Development: A Cause–Effect Analysis
Source: Int J Mol Sci. 2024 Sep 20;25(18):10130. doi: 10.3390/ijms251810130 (PMC11432622; doi:10.3390/ijms251810130)

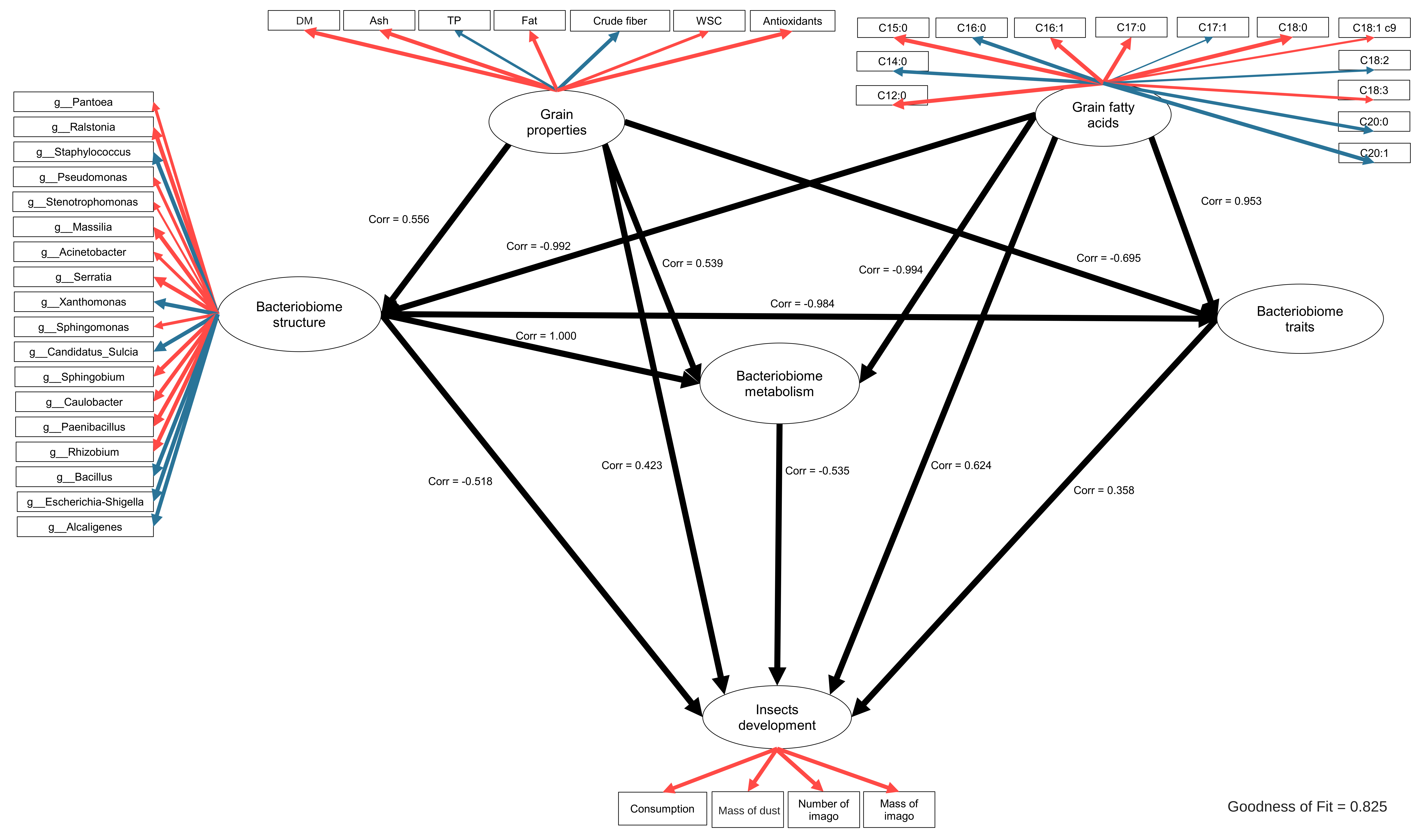

Supplement: Supplementary file 1 [file ijms-25-10130-s001.zip › Supplementary File(s)/Figure S1.png]

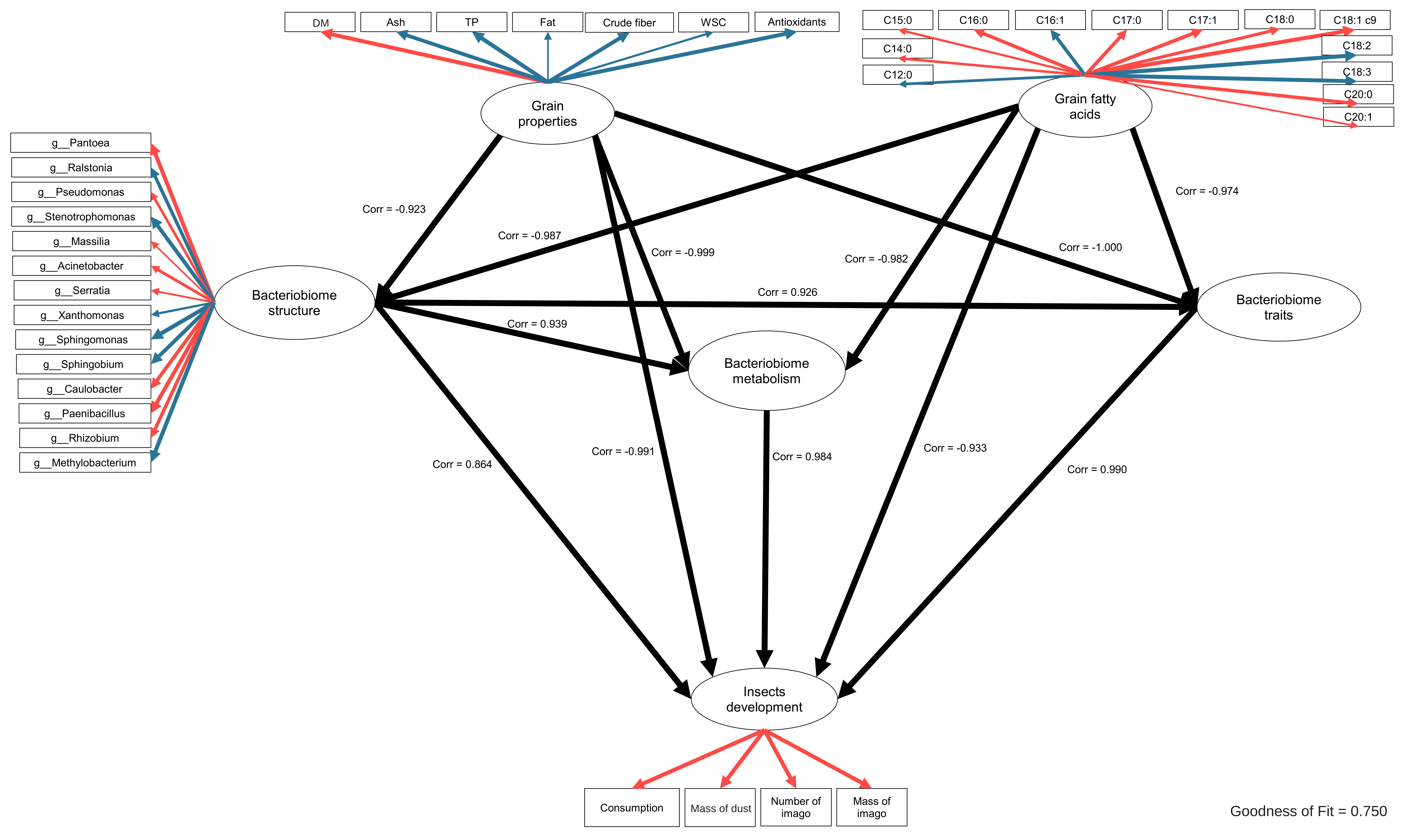

Supplement: Supplementary file 1 [file ijms-25-10130-s001.zip › Supplementary File(s)/Figure S2.png]

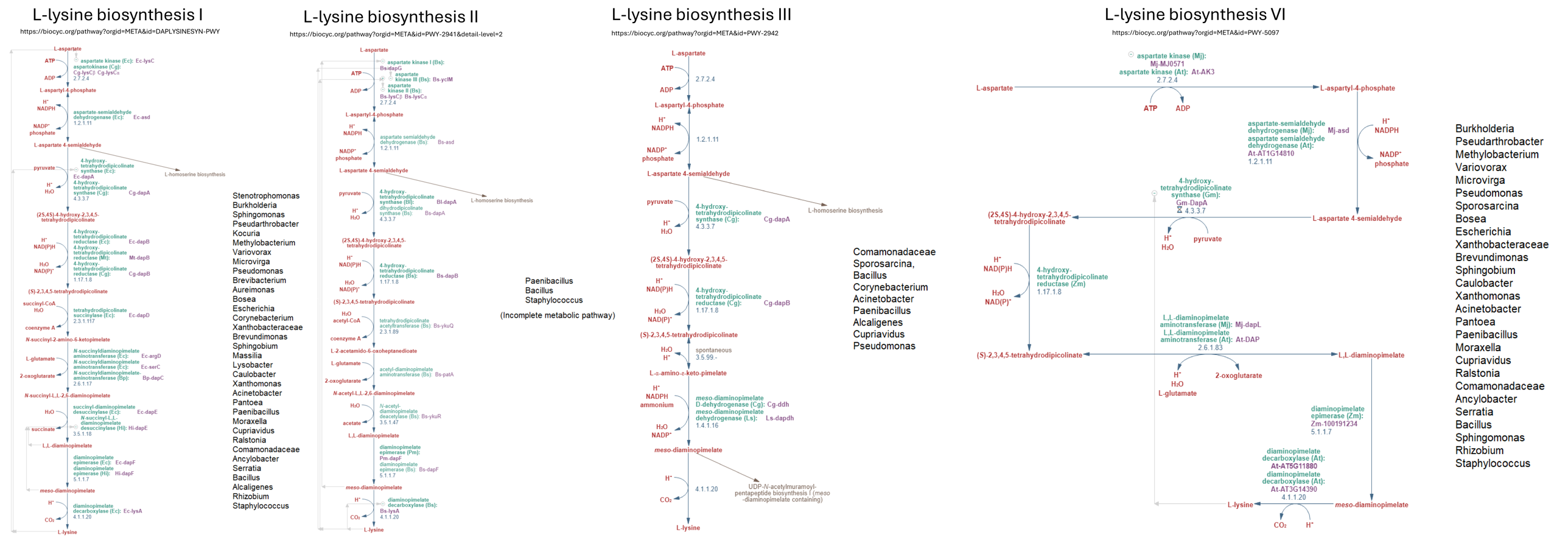

Supplement: Supplementary file 1 [file ijms-25-10130-s001.zip › Supplementary File(s)/Figure S3.png]
